# A Personal Respirator to Improve Protection for Healthcare Workers Treating COVID-19 (PeRSo)

**DOI:** 10.3389/fmedt.2021.664259

**Published:** 2021-06-10

**Authors:** Paul T. Elkington, Alexander S. Dickinson, Mark N. Mavrogordato, Daniel C. Spencer, Richard J. Gillams, Antonio De Grazia, Sebastian Rosini, Diana J. Garay-Baquero, Laura E. Diment, Nitin Mahobia, Alexandra Mant, Tom Baynham, Hywel Morgan

**Affiliations:** ^1^School of Clinical and Experimental Sciences, Faculty of Medicine, University of Southampton, Southampton, United Kingdom; ^2^Institute for Life Sciences, University of Southampton, Southampton, United Kingdom; ^3^NIHR Biomedical Research Centre, University Hospital Southampton NHS Foundation Trust, Southampton, United Kingdom; ^4^Mechanical Engineering Department, Faculty of Engineering & Physical Sciences, University of Southampton, Southampton, United Kingdom; ^5^School of Electronics & Computer Science, Faculty of Engineering & Physical Sciences, University of Southampton, Southampton, United Kingdom; ^6^Department of Infection, University Hospital Southampton NHS Foundation Trust, Southampton, United Kingdom; ^7^INDO Lighting Ltd., Southampton, United Kingdom

**Keywords:** COVID-19, respiratory infections, personal protective equipment, PPE, powered air purifying respirator, PAPR

## Abstract

**Introduction:** SARS-CoV-2 infection is a global pandemic. Personal Protective Equipment (PPE) to protect healthcare workers has been a recurrent challenge in terms of global stocks, supply logistics and suitability. In some settings, around 20% of healthcare workers treating COVID-19 cases have become infected, which leads to staff absence at peaks of the pandemic, and in some cases mortality.

**Methods:** To address shortcomings in PPE, we developed a simple powered air purifying respirator, made from inexpensive and widely available components. The prototype was designed to minimize manufacturing complexity so that derivative versions could be developed in low resource settings with minor modification.

**Results:** The “Personal Respirator – Southampton” (PeRSo) delivers High-Efficiency Particulate Air (HEPA) filtered air from a battery powered fan-filter assembly into a lightweight hood with a clear visor that can be comfortably worn for several hours. Validation testing demonstrates that the prototype removes microbes, avoids excessive CO_2_ build-up in normal use, and passes fit test protocols widely used to evaluate standard N95/FFP2 and N99/FFP3 face masks. Feedback from doctors and nurses indicate the PeRSo prototype was preferred to standard FFP2 and FFP3 masks, being more comfortable and reducing the time and risk of recurrently changing PPE. Patients report better communication and reassurance as the entire face is visible.

**Conclusion:** Rapid upscale of production of cheaply produced powered air purifying respirators, designed to achieve regulatory approval in the country of production, could protect healthcare workers from infection and improve healthcare delivery during the COVID-19 pandemic.

## Introduction

With the global SARS-CoV-2 pandemic, there is an urgent need to protect healthcare workers (HCW) from infection; healthcare workers are “every country's most valuable resource” (1). Healthcare workers are at a significantly increased risk of infection with SARS-CoV-2, and initial infectious inoculum is likely a determinant of disease severity (2). For example, 11% of UK staff showed evidence of COVID-19 in a large UK teaching hospital after the first wave (3) and 20% of HCWs treating people with COVID-19 in Italy were infected in the first wave (4), even in the context of a relatively good supply of personal protective equipment (PPE) compared to low income countries. HCW infection leads to significant staff absence at critical points during the pandemic, and unfortunately will result in mortality, resulting in prolonged impact on healthcare systems particularly in resource-poor settings.

In the early response to pandemics where vaccination is not available, PPE plays a major role in control programs. Analysis of the efficacy of protective equipment during the SARS outbreak in hospital settings demonstrated that failure to implement necessary barrier precautions was responsible for nosocomial transmission (5). Current standard PPE for COVID-19 is disposable, and there are issues around suitability and waste (6). HCWs may receive limited PPE fit testing, training and provision due to the disruption caused by the COVID-19 pandemic. In the first countries to experience a peak of transmission, such as Italy, supplies of PPE ran out in some hospitals, and UK guidelines on how PPE should be used required updating during the pandemic's first wave (7). In addition, transmission of infection often precedes symptoms, and so undiagnosed patients may exacerbate transmission in healthcare settings (2).

PPE is selected by considering the route of disease transmission (e.g., airborne, droplet or contact). SARS-CoV-2 infection can be transmitted by both large droplets and smaller aerosolised particles with a substantial airborne component (2, 8). Respiratory Protective Equipment (RPE) that can be continuously worn provides protection against airborne pathogens, as well as a physical barrier to avoid transmission from surfaces by preventing facial touching. The three main RPE categories currently in use are filtering facepiece respirators (FFR, i.e., N95/FFP2 or N99/FFP3 masks), full facepiece respirators (which features an integrated screen or visor fitted around the face), and powered air purifying respirators (PAPR).

Filtering facepiece respirator masks are designed to protect HCWs from infection whilst treating patients, as distinct from surgical masks which are intended to protect others from the wearer (9). Reported limitations of N95/FFP3 masks include interface pressure between the mask and face, thermal discomfort and skin tissue injury after long-term use, impairment to breathing, failure of the face-mask seal whilst talking and recurrent fit testing failure for certain face shapes, effectively removing ~5% of the workforce (10–13). The standard that defines FFR efficiency allows 5% inward leakage maximum, and 1% aerosol filter penetration by 0.02–2 μm (median 0.6 μm) particles (14). Full-face respirators and respirators with hoods offer greater respiratory protection and more facial coverage against splash or accidental touching than FFRs (15). Elastomeric full-face respirators require a tight seal, and so may cause skin discomfort, rashes and oedema if worn for long periods. In contrast, hood-type PAPR devices are reported to be more comfortable by delivering a continuous airflow without requiring tight fitting (16). This type of respirator uses a fan and filter to deliver clean air to the user, with sufficient air flow rate that the air pressure inside the hood remains higher than outside throughout the breathing cycle. This produces a steady net outflow of air from the hood around the loose neck seal and through any seams. Furthermore, the hood prevents inadvertent facial touching by the user, avoids issues of compromised fit between masks and goggles, and removes the need to change the PPE repeatedly, thereby improving the clinician's efficiency. Off-ear hoods may provide similar protection while using a soft elastic seal under the chin and at the sides of the face, and permit the use of stethoscopes and telephones. Reusable PPE will eventually be more cost effective and environmentally sound than single-use items.

To address shortcomings in PPE availability, we developed a simple PAPR made from inexpensive and widely available component parts. The prototype has few manufacturing steps so that derivative versions can be developed for use in low resource settings with minor modification to protect healthcare workers during the COVID-19 pandemic.

## Methodology

**Figure 1:**
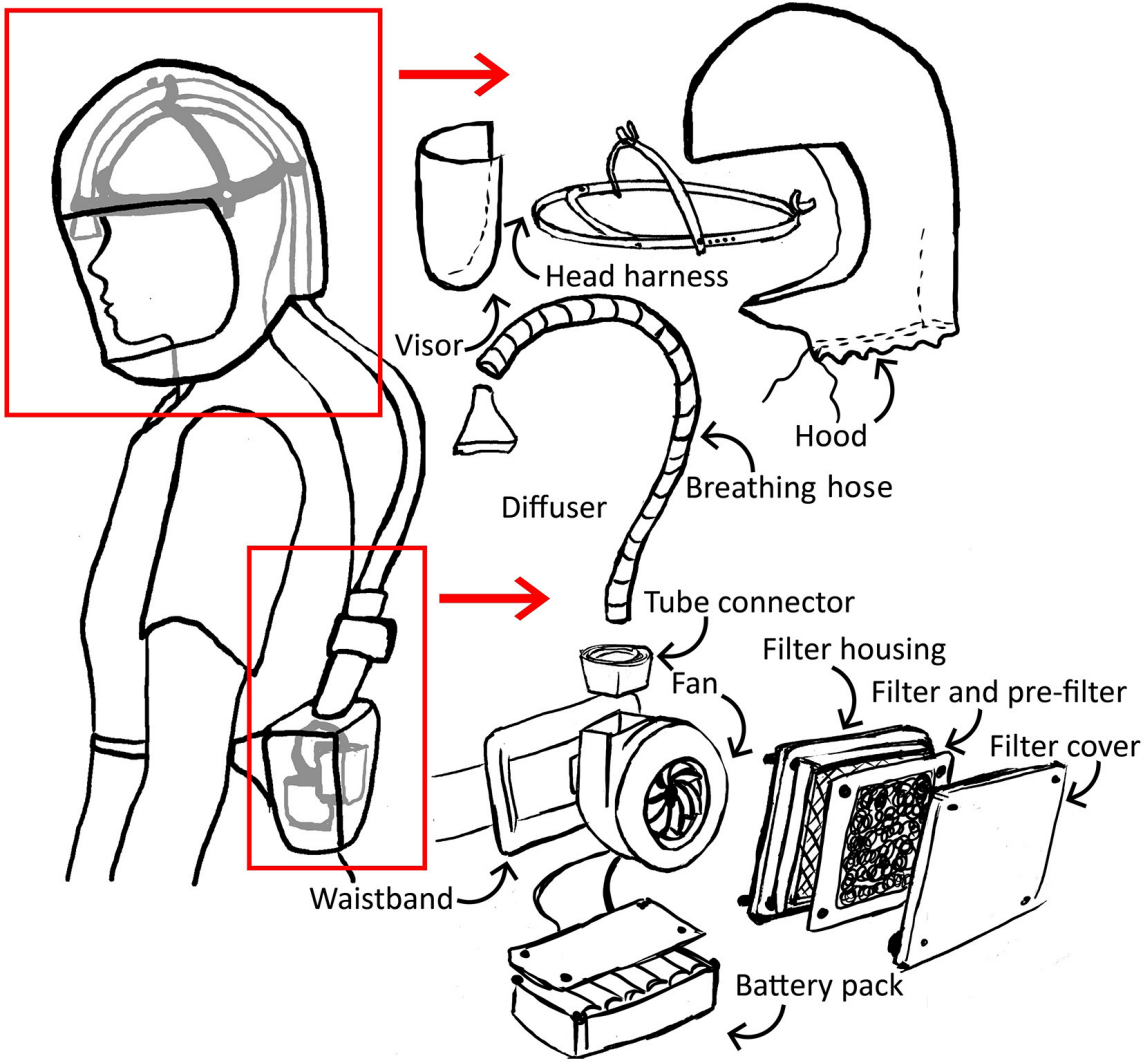
Sketched system diagram for a simple powered air purifying respirator (PAPR).

The initial concept for a simple respirator incorporated a filter, blower fan and power supply worn on a belt pack, with air delivered by a breathing hose to the head harness, within an enclosed, loose-fitting hood with visor (Figure 1).

Clinical staff at University Hospital Southampton NHS Foundation Trust, UK, provided the initial user needs outline for a PAPR respirator designed to replace FFP3 masks and surgical masks, with respiratory- and droplet splash-protective capability to the Standard of BS EN12941 (17). The initial design brief proposed that the respirator be made from inexpensive and widely available component parts, to address supply chain problems with commercial devices. It had to be comfortable to wear for an 8 h shift with one break, and be stable during use, including walking around the hospital, bending and performing cardio-pulmonary resuscitation (CPR). The hood and visor needed to provide full vision for the user and minimize hearing impairment, as well as providing patients and colleagues with full face visibility. The respirator needed to be reusable over the duration of the pandemic, for a minimum period of 6 months, and easily decontaminated and cleaned. Practically, it needed to be easily put on and taken off (“donned and doffed”), potentially with one assistant. The components should ideally be low profile, to prevent snagging on surrounding equipment, and the belt-mounted unit worn over medical “scrubs” clothing but underneath a front-loading gown or apron, and covered with a cleanable cover or cowl. Contributors to these requirements included staff with experience of using other commercially available PAPR devices, and thus they represent the specific requirements of a PAPR device for healthcare workers during the COVID-19 pandemic. Only some of these requirements are expressed in the general PAPR Standard (17).

These user needs were then translated into the technical specification expressed in Table 1.

**Table 1:** The PeRSo open specification.

**Component**	**Description & example embodiment**
Waterproofing/Cleaning	• System must be water resistant and tolerant to common cleaning agents, such as 1,000 ppm chlorine, for example by spraying a cloth with solution and wiping down. • Estimated Ingress Protection target of IP-65 or IP-67.
Filter & housing	• High-Efficiency Particulate Air (HEPA) filter (18), ideally H13 or H14; potentially sourced from a vacuum cleaner, dehumidifier or equivalent. • Prefilter for large particles to maximize HEPA lifetime. • Robust protective cover to avoid mechanical damage, puncture and moisture ingress during cleaning.
Blower	• Centrifugal fan delivering >170 l/min (19) at the system backpressure, depending on choice of filter, breathing hose and tubing in headgear. Ideally medical grade.
Power supply	• Rechargeable batteries or battery pack. • Minimum 4 h runtime, ideally >12 h. • On/off switch, protected for cleaning and to avoid accidental power-off. • A low-power warning with >15 min runtime.
Blower unit housing and waist band or backpack	• Airtight assembly to mount filters to fan; runner's belt bag or backpack. • Covered using a cleanable or disposable cowl.
Breathing hose	• Low mass and high flexibility to provide minimal impedance to head movements and strain on neck muscles. • Either cut to length for user or length-adjustable. • Ideally medical grade material e.g., polyurethane, PVC. • Typical inner diameter 25–32 mm.
Connectors	• Screw or bayonet type; potentially with internal helix matching thread formed by reinforcement on breathing hose. • Ideally universal connection between blower unit and breathing hose so blower unit can be exchanged between users between shifts after appropriate decontamination.
Head harness	• Comfortable use for an 8 h shift, avoiding direct contact with facial skin. • Adjustable to different head sizes and shapes. • Supporting breathing hose. • Easily attachable and removable mounting of hood.
Hood	• Hydrophobic material, (e.g., Tyvek, Vent3 polypropylene breather membrane). • Latex-free. • Taped or stitched transparent polymer visor (e.g., PVC, polycarbonate), optically clear and resistant to fogging or creasing. • Designed for single-user, with label showing user's name and role to aid identification. • For a PAPR device, the hood, face/neck seal and tube connector do not need to be air-tight, as the clean airflow and positive pressure prevents ingress of particles.

### Candidate Component Types

The reasons for selecting components are provided below, although available products may vary considerably between settings and budgets. Key components are described to expand details provided in Table 1.

Excellent bacterial and viral filtering performance has been demonstrated through use of HEPA (High Efficiency Particulate Air) filters (20). HEPA filters are usually produced from microfiber glass. The HEPA filter standard specifies removal of at least 99.95% (H13 grade) and 99.995% (H14 grade) of particles with diameters >0.3 μm, near to the Most Penetrating Particle Size (MPPS) (18). Various mechanisms play a role in the filtration efficiency such as the depth of the filter layer, the density of the fibers, and the velocity of the gas to be filtered, and these efficiencies are tested and valid at the designer's specified nominal flow-rate. In most general terms, HEPA filters impede larger particles by interception and impaction on filter fibers and smaller particles by diffusion processes. Both mechanisms are potentially relevant for PAPRs: particles in a continuum of sizes (2) ranging from 0.01 to 500 μm are generated in increasing volume during breathing, speaking, coughing and sneezing (21, 22) and coronavirus virions are reported to range from 0.05 to 0.15 μm in diameter with average diameters around 0.08 to 0.09 μm (23–25). HEPA filters capture a wide range of particles efficiently, with a relatively low pressure drop, and these characteristics make HEPA filters an optimal option for use in respirators that are intended to protect against COVID-19 airborne infection transmission.

Centrifugal fans are preferable to axial fans, because they can generate higher pressure to overcome the pressure drop across the filter and the breathing hose. They are also quieter and deliver a steadier flow rate with reasonably low power consumption.

A practical means to address logistical and supply issues, and provide flexible implementation of this specification in low resource settings, we propose universal connectors between the components within the system. Universal screw-fit or bayonet connectors may allow assembly of different blower fans, breathing hose, and headgear. Furthermore, connecting the hood's visor to the head harness using adhesive Velcro allows easy hood removal for cleaning and replacement, and use of different hoods for different levels of protection. For example, anesthetists performing aerosol-generating procedures such as intubation may prefer to use hoods providing greater coverage of the neck and shoulders compared to a head cover for a physician reviewing patients on a ward round.

### Prototype Assembly

**Figure 2:**
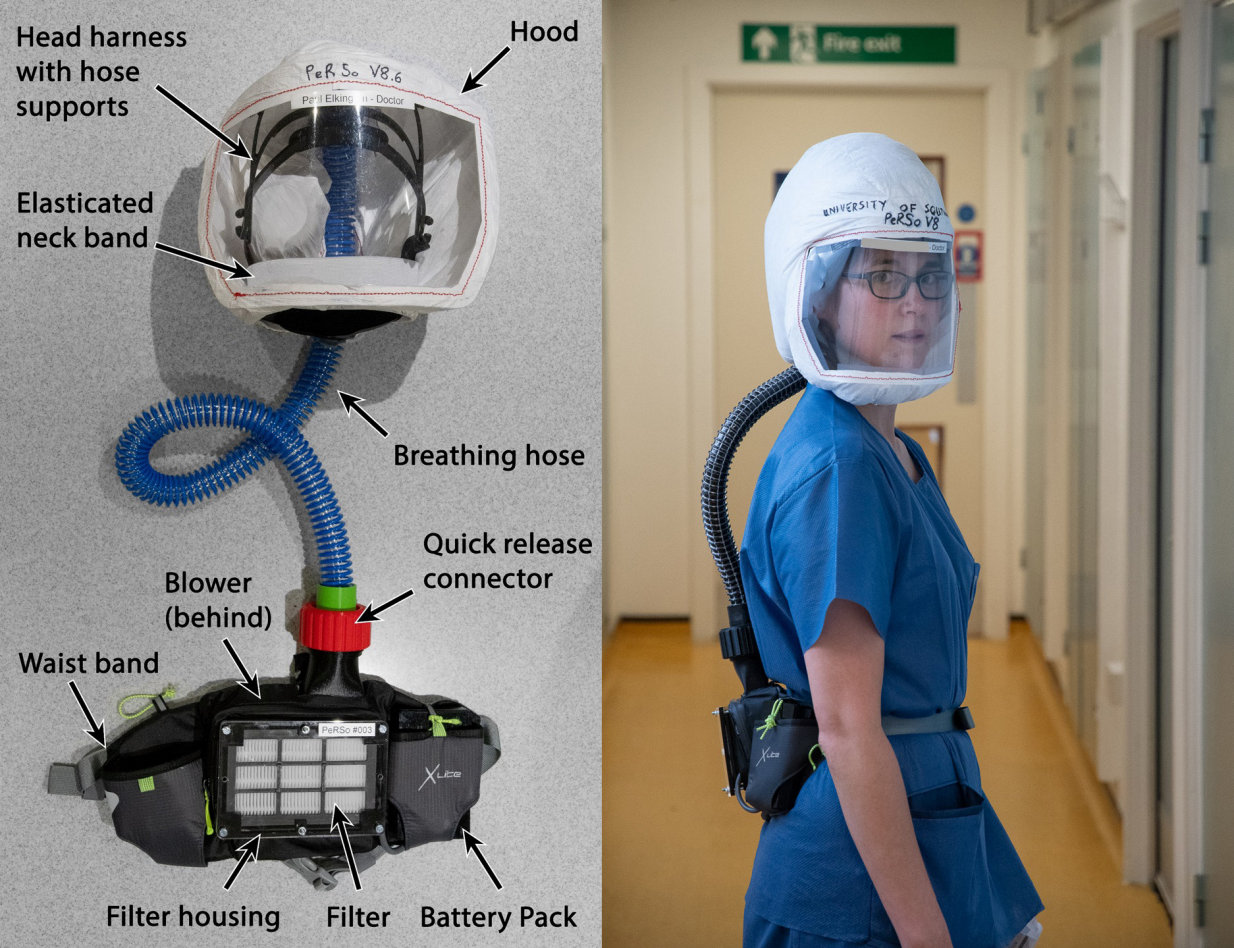
Prototype PeRSo Respirator system layout **(left)**, and donned by a user to show ergonomics **(right)**.

**Figure 3:**
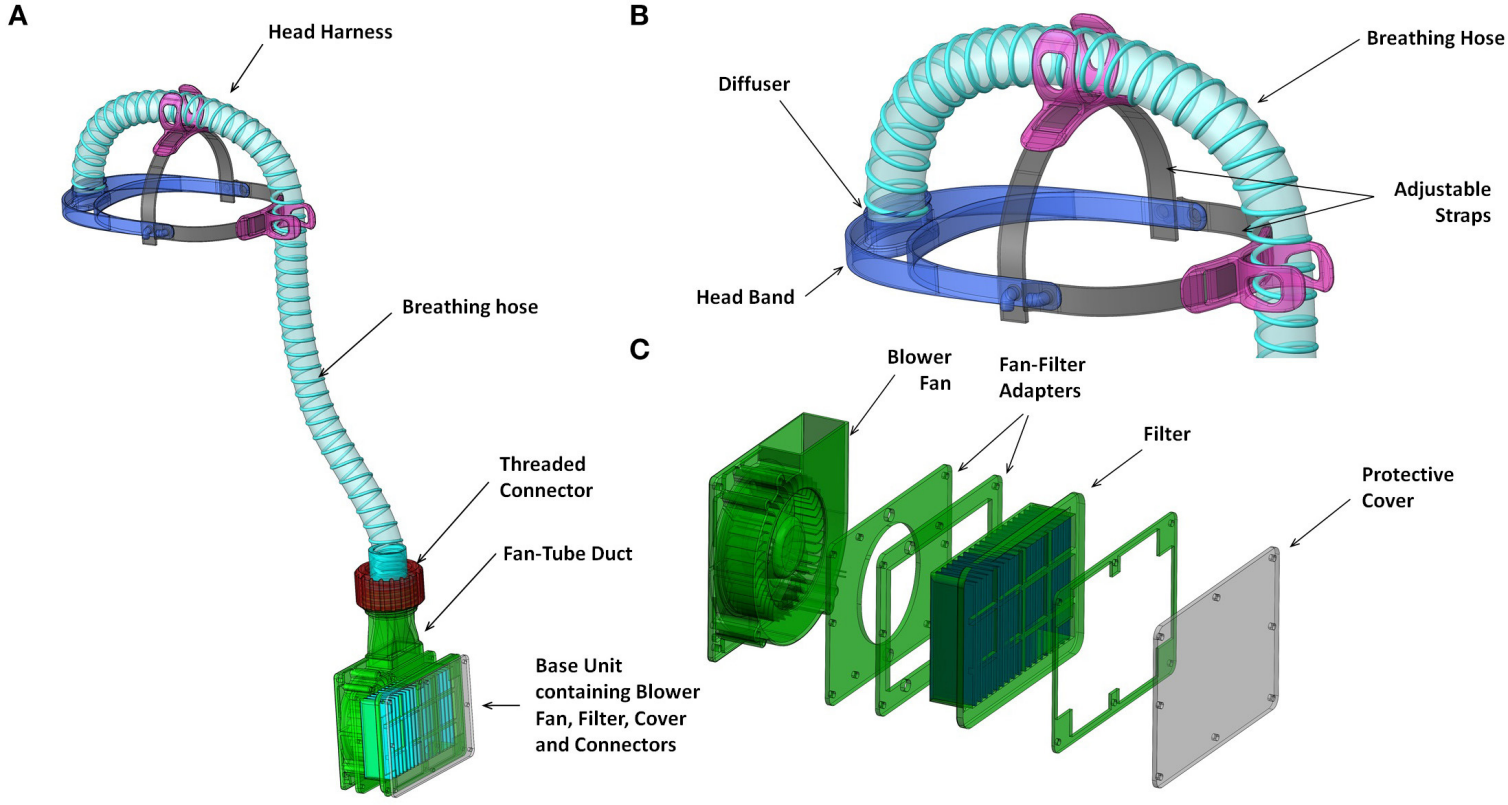
Renderings of key functional components **(A)** overall system with hood removed; **(B)** head harness including head band and attachments to breathing hose; components 3D printed in first prototype; and **(C)** blower unit assembly including fan-filter interface stack and protective cover from laser cut acrylic.

The prototype PeRSo device (Figure 2) was assembled using a widely available 10W 12V centrifugal fan (PMB1212PLB2-A (2). GN, SUNON, Taiwan), a HEPA filter sold as a vacuum cleaner spare (85667205, John Lewis & Partners, UK), corrugated plastic breathing hose, and a customized harness designed by McLaren Racing Ltd (Figures 3A,B). For the prototype, this featured a headband 3D printed by filament deposition modeling (FDM) in ASA (Acrylonitrile styrene acrylate) material, with adjustable rubber straps, and breathing hose clip and duct components 3D printed in chopped carbon fiber filled Nylon (CF12) and ABS (Acrylonitrile butadiene styrene). Alternatively, the headband can be made using components removed from an off-the-shelf brow guard or builder's hard hat, which provides an adjustable, comfortable support for the breathing hose and visor.

The fan and filter housing was produced from a stack of laser cut 3 mm acrylic sheet that provided an air-tight interface (Figure 3C). The interface between the fan and acrylic was made airtight with standard silicone sealant. A threaded connector was designed to join the breathing hose and fan outlet, 3D printed by FDM in PLA (polylactic acid). These duct components, which require airtightness, were printed using a 0.4 mm nozzle, 0.3 mm layer height, and 50–60% infill. The breathing hose was mounted on the headband with 3D printed clips, and its open end crimped at the forehead in order to act as a diffuser to distribute the airflow over the visor and the user's face. A standalone diffuser component could be used, but the crimped hose was sufficient to diffuse the air and was simple to manufacture. A loose-fitting hood was made from Tyvek material cut from a widely available protective coat, with a 0.25 mm PVC visor and elasticated neck band. The fan, filter and power assembly unit was worn around the waist in a commercially available runner's belt-bag. The finished assembly weighed 1.39 kg, split as 0.95 kg from the belt-mounted components and 0.44 kg for the head-mounted components and breathing hose. The total estimated cost of parts and materials was in the range of £69–£89 (Table 2).

**Table 2:** Estimated cost of parts and materials.

**Component/material**	**Est. cost**
Fan	£15
Filter	£5
Batteries	£13
Breathing hose	£5
Runner's belt bag	£13
Hood (scratch made)	£5
Hood (off-the-shelf)	£25
3D printed parts	£5
Misc (acrylic, sealant, neoprene strap, nuts, bolts)	£8
**Total (w/scratch-made hood)**	**£69**
**Total (w/off-the-shelf hood)**	**£89**

^£^*Represents GBP/Pounds Sterling*.

### Patient and Public Involvement

As mentioned above, a wide variety of clinical and non-clinical staff at University Hospital Southampton NHS Foundation Trust were consulted at two points in the reported work, to inform both the development of the respirator specification, and to provide feedback upon the usability of the prototype respirator. These took the form of informal group conversations, around eight general questions (Appendix), following a general Patient and Public Involvement (PPI) framework. Patients provided feedback upon the appearance of the prototype respirator after the initial design process. These took the form of informal verbal feedback on the wards of University Hospital Southampton NHS Foundation Trust to individuals wearing prototype respirators.

For more thorough tests of filtration, air tightness and CO_2_ concentration within the hood, ethical approval was granted by an institutional committee (ERGO/FEPS/61406).

## Results

### Filtration and Air-Tightness Tests

A qualitative filtration test was conducted according to ISO16975-3 (26), using the 3M FT-30 & FT-32 solution and spray apparatus, which is routinely used to test performance of standard FFP2 and FFP3 masks, to assess the efficacy of filtration and identify leakage. Tests took ~5 min and were conducted at the point of fitting and repeated in some users at the end of use, including after one >8 h shift. The nebulised test aerosol was sprayed directly onto the HEPA filter and also around it, and no ingress was detected by three different assessors, indicating absence of droplet penetration through the filter. Similarly, no droplets penetrated when the nebuliser aerosol was directed at the hood or connectors. A positive control test was then conducted by switching the blower unit off and spraying the solution inside the hood, which gave positive results in all three assessors, confirming they could taste the test solution. While the spray test represents larger droplets, the test was repeated with cigarette smoke blowing directly at the hood, which has much finer particle size (averaging 0.09–0.3 μm, with many smaller particles and volatile organic compounds in the distribution) (27). Again, no penetration into the hood was detected by the wearer.

### Respirator CO_2_ Concentration Tests

A capnograph (Capnocheck Sleep, Smiths Medical Inc., USA) was used to measure the CO_2_ concentration within the hood for three individuals, with the capnograph tube passing through the hood seal and taped on the cheek close to the mouth. A protocol was designed to represent the physical exertion representing use in a hospital setting, and the undesired scenario of filter blockage and if powered off. The wearer was asked to: (i) breathe normally, (ii) breathe heavily, and (iii) run on the spot, each for 1 min. Then (iv) the respirator blower unit was placed in a loosely sealed bag to replicate filter blockage, and then (v) the fan was switched off. In both scenarios the wearer was asked to breathe normally for 1 min. Finally the respirator was switched back on. Tests were repeated three times. A pass criterion was defined at 1% CO_2_ concentration during normal breathing (17), and the tests were repeated using a standard surgical mask for comparison. CO_2_ concentration (Table 3) was below the 1% pass criterion for normal breathing, and for heavy breathing in 8/9 tests, and peaked at 3.5% in vigorous activity and when the blower unit was enclosed and when switched off. In all cases CO_2_ concentration returned to 0% within 10 s of normal operation.

**Table 3:** Peak CO_2_ build-up levels in the prototype respirator and surgical mask.

	**Mean (s.d.) % CO**_**2**_ **concentration, accuracy ± 0.3%**	
	**Prototype respirator**	Surgical mask^*^
Ambient air (control)	0 (0)	0 (0)
Normal breathing	0 (0)	6.0 (0.4)
Breathing heavily	0.8 (0.2)	3.5 (0.4)
Running on the spot	2.4 (0.3)	6.2 (0.4)
Normal breathing with respirator in loosely sealed bag	2.0 (0.3)	n/a
Normal breathing with respirator switched off	2.3 (0.8)	n/a

* *Note that these values represent peaks observed during sinusoidal concentration changes with the breathing cycle, whereas the values in the respirator hood were sustained*.

### Microbiological Filtration Efficacy

**Figure 4:**
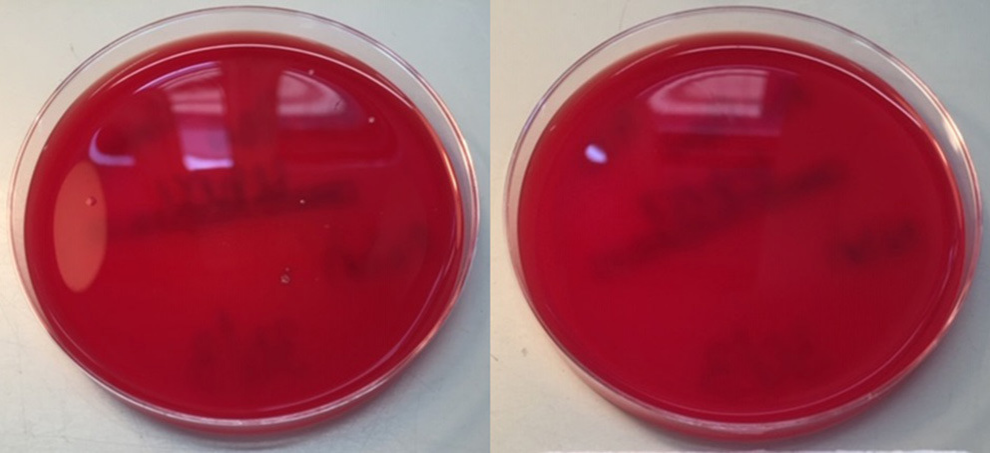
Settle Control (left) and Test (right) plates, showing 10 and 0 colony forming units of bacteria, respectively.

A quantitative assessment of microbiological air filtration was performed using passive sampling with settle plates according to ISO14698 (28), by placing a Columbia blood agar culture plate within the respirator hood. This procedure was repeated 7 times, once with a settle plate placed outside as a positive control, and in the other 6 with control tests inside a respirator hood with the filter removed. The respirator was activated for 6 min to deliver the standard-specified 1,000 liters of room air through the blower unit onto the agar plate. The culture plate was then incubated for bacterial growth for 48 h at 37°C. The open settle control plate had ten colonies of bacteria, and the six no-filter controls had 1–3 colonies (median 2). No culture plates inside the respirator hood with a filter had any growth, equivalent to zero CFU (colony forming unit) per cubic meter of air (Figure 4). This indicates that the respirator was performing as intended in terms of bacterial filtration from ambient air (*p* = 0.0013), to an equivalent standard expected from an operation theater environment (29).

### Healthcare Worker Feedback

Six physicians, and 2 nurses and healthcare assistants (5F:3M) provided user feedback on PeRSo prototypes whilst working standard shifts on National Health Service (NHS) wards. Doctors commented on the advantage of not needing to change PPE frequently between patients, which made delivery of care much more efficient. A perception of greatly improved protection was reported when working on wards where surgical face masks are the standard Public Health England (PHE) guidance, such as COVID-19 confirmed general inpatient wards. Numerous reports stated that the flow of air down the face was much more comfortable than an FFP3 mask for use over a full shift. Other user comments included themes around:

- Comfort and Endurance: “Masks are hot and this was cool and not claustrophobic. Normal mask straps make the top of my ears sore but this doesn't do that.”; “Brilliant! Used for over 8 h yesterday and battery still going strong.”
- Confidence: “Good. Felt safe”; “Excellent sense of protection whilst doing procedures.”
- Communication: “Was easy (in some ways better than masks) to communicate”; “Compared with mouth/nose mask, nice to see faces, helps with communication.” One drawback noted was that hearing patients talking quietly was harder, but to compensate the wearer was more confident to get closer to hear.
- User Experience: “Removal of PPE is often a high risk point for accidental nosocomial infection. By not having to change PPE all the time, I felt safer and was able to deliver my clinical duties much more effectively.”
- Scope for Improvement: Some tasks were more difficult, notably manual blood pressure monitoring by nurses using a stethoscope, outside critical care areas where heart monitors are used.

People also gave feedback on usability, with regard to:

- Donning: “Harder because of wearing glasses but not a problem. Long hair needs to be in ponytail, i.e., flat, to fit hood on and fit the straps.”; “No issues putting the mask on”; A drawstring version of the hood was reported to be easier to don than an elasticated version, for people who wear glasses.
- Comfort: “I liked the feeling of fresh air coming down over my face. It was very much more comfortable than a standard face mask which I find get hot very quickly.” “I wear glasses and they didn't steam up unlike a standard mask.” “Very comfortable until after 6 h I found the forehead strap falling down, and denting my skin for a while after. I will try to readjust the fitting next week. Maybe the thick sponge foam material used in cycling helmets would help?” “I did feel my visual field was limited at times however the material on the hood was light, the filtered air was lovely and a hair net would help with your hair as it kept getting in my eyes. I didn't feel overly hot wearing it.”
- Doffing: “Very easy.”; “More difficult when you wear glasses, and hair gets in the way but the second time we did this was a bit easier as we knew what to do.”; “Easy. My technique was to have a buddy (wearing apron mask and gloves) ready with Clinell wipes to wipe down the hood, visor, pipe and belt. Once this was done I dropped the whole lot in the plastic box.”

## Discussion

We report the development and validation of an Open Specification prototype for a personal respirator that delivers HEPA filtered air to a hood to protect healthcare workers during the COVID-19 pandemic. The design is relatively simple, components are inexpensive and where newly designed components were required, considerations for mass production have been incorporated. We have made the design files and a step-by-step guide openly available to assist manufacture, and produced further information for individuals working in low-resource settings (https://www.southampton.ac.uk/persodw) (30). Although some parts were 3D printed for the prototype head-harness, these could equally be replaced by using the internal component of a builder's hard hat and Velcro straps. Preliminary tests and initial user evaluation have been very positive. However, moving from established healthcare PPE use to a new system requires a careful evaluation of risk.

During the pandemic, the UK Government published new guidance clarifying approval of PPE (31), which draws on BS EN 12941 for loose fitting respiratory protective equipment (17). This standard includes a wide range of verification tests beyond filtration efficacy, such as hose and coupling strength, hood leakage, breathing resistance, field of vision, noise, and resistance to flame. The UK Government's Department for Business, Energy and Industrial Strategy has instructed via the Office for Product Safety and Standards guidance to the Health and Safety Executive (HSE), to engage in “regulatory easing” for new market entrants of non-novel PPE. The intention was that this temporary change in the regulatory regime for respiratory protective equipment (designed and tested to BS EN 12941) can be translated more swiftly from prototype to product while testing is ongoing.

The testing of the prototypes has to date been over the short term, and prior to established use in healthcare settings there is a requirement to demonstrate safety and the duration of effectiveness and durability of the device and its component parts for longer periods. The first manufactured version inspired by our work has now been approved by the HSE, and two versions (PeRSo1 and PeRSo3, INDO Lighting Ltd., Southampton, UK) have been certified by the British Standards Institution (certificates CE 728691, 728692 and 740159) following full testing to BS EN 12941 (17). In our experience, hoods require replacement after approximately 2 months, and the local use protocol for these devices includes filter replacement at 6 monthly intervals. Durability of other components is harder to assess. Lithium ion batteries may have a useful life of 500 cycles, representing a year of use, but this depends on care during use and charging. Fan manufacturers also rarely publish mean time to failure (MTTF) and 10% failure lifetime (L10) data as they are likely to depend on the particular use case. The endurance of 3D printed parts and their permeability might be affected by serial cleaning and decontamination cycles, but these measures might be verified by the physical tests in BS EN 12941 (17) or ingress protection tests to BS EN 60529 (32). In cases of more extreme use, such as elevated temperature, filtration efficacy should not be affected, but with high humidity additional verification may be required.

The prototype respirator should capture a greater percentage of airborne droplets relative to FFP2/N95 masks as it incorporates a HEPA filter, which is highly efficient, and it does not have issues with loss of seal that often occur with standard face masks. Therefore, PAPRs should reduce an individual's exposure to viral particles. Furthermore, a seal failure rate of 18% is reported for FFP3 masks during activity such as cardio-pulmonary resuscitation (33), whereas PAPRs have a 0% failure rate during simulated chest compression (34). Additional advantages over mask-type FFR devices are resolving the 5% staff exclusion rate due to persistent mask fit testing failure, greater comfort and skin health, cooler temperature, improved communication (except perhaps by telephone), inherent eye protection from droplets, avoidance of the user inadvertently touching their face, and lower breathing resistance (34, 35). In addition, UK HSE guidance states that tight-fitting masks should not be used for continuous wear for over 60 min (36), which is consistently exceeded in healthcare settings. The primary disadvantages of any full hood-type respirator is that over-the-head aprons can take time to put on, and a stethoscope cannot be used if a neck-length hood is worn. Consequently, feedback showed that sleeker hoods that leave the ears exposed are preferred in most settings, and these have now been introduced.

Hood type respirators require different protocols for clinical use to FFP2 and FFP3 masks. New procedures are required for putting the device on and taking it off, with healthcare staff training necessary to avoid increasing the risk of viral exposure (37). “Doffing stations” for removal of respirators could be established where healthcare workers are assisted with decontamination by wiping down with chlorine, performed by support staff also wearing PPE. Without these usage protocols, personal respirators may not be protective, and members of the general public and untrained clinicians should not use these devices as they may inadvertently contaminate themselves or increase transmission from asymptomatically infected individuals (2).

Beyond the necessary approvals and regulations for providing these devices, developers should also be aware of intellectual property and copyright issues if they are directly reverse-engineering existing devices. In the present work, we have avoided direct reverse-engineering, motivated by developing a lower-cost accessible device made from off-the-shelf components where possible that can be manufactured in low-resource settings.

To the best of our knowledge, powered air respirators have not been widely implemented globally to protect healthcare workers during the COVID-19 pandemic, primarily because existing, certified devices were expensive (typically £600–£1,100) and produced to specifications which cover a wide range of scenarios, not all of which are required for healthcare use. In addition, most were unavailable in the UK during the first wave of COVID-19 infections. Lower cost devices were available at large online marketplaces but had unclear certification status. We propose that the PeRSo, with a deliberately simple design and low cost [production variant PeRSo1 (INDO Lighting Ltd.) was made available at £225 in volume], could be deployed within a tertiary care environment to reduce the supply chain issues related to standard PPE, improve the comfort and usability for wearers, and enhance the patient experience. Going forward, it will be necessary to undertake full evaluation of user risk vs. health economic benefits, the effect on staff well-being, irritation and infection rates, and the overall effect on inpatient standardized mortality. However, performing such studies is challenging when the epidemiology of infection is rapidly changing, as the effect of deployment is likely to be obscured by the local incidence of infection, which varies rapidly. User evaluation will require different assessment relative to cultural settings, especially in consideration of lower and middle income countries. Significant obstacles to widespread deployment are financial, supply chain and mass manufacture, logistical implementation and cultural. Furthermore, for each center a detailed implementation plan will be required including putting on and removal protocol, battery recharging, cleaning and maintenance such as periodic filter replacement, to ensure ongoing protection during the pandemic and over the time period of reuse. These are essential considerations but beyond the scope of this specification and prototype report.

## Conclusion

The PeRSo provides multiple potential advantages over current PPE provision. As it is reusable, deployment can address supply chain problems especially in low resource settings where PPE shortages are most acute. In addition, PeRSos resolve face fit testing failure for FFP3 masks and leakage during use, provide visibility of the whole face, ease of movement between patients without changing PPE, and improve overall comfort. Many of the components are commercially available and relatively cheap, and parts can be mass produced. Major governmental investment has been made in manufacturing ventilators and developing vaccines, and we propose that the next global effort should aim for prevention of infection. Fully developed, the PeRSo device could reduce global mortality by improving efficiency of healthcare staff in caring for patients, and concurrently reducing staff illness and mortality.

## Data Availability Statement

The datasets presented in this study can be found in online repositories. The names of the repository/repositories and accession numbers can be found below: https://doi.org/10.5258/SOTON/D1300.

## Ethics Statement

The studies involving human participants were reviewed and approved by University of Southampton institutional ethics committee ERGO (Ethics and Research Governance Office). The patients/participants provided their written informed consent to participate in this study. Written informed consent was obtained from the individuals for the publication of any potentially identifiable images or data included in this article.

## Author Contributions

PTE: literature search, study design, data collection, data analysis, data interpretation, and writing. ASD: literature search, figures, technical design, data collection, data analysis, data interpretation, writing, and underlying data verification. MNM: figures, technical design lead, data collection, data analysis, and data interpretation. DCS: literature search, technical design, data collection, data analysis, and data interpretation. RJG: figures, data collection, and data analysis. ADG: data collection and data analysis. SR: figures, data collection, data analysis, and underlying data verification. DJGB: literature search and writing. LED and NM: data collection and data analysis. AM: literature search, writing, data collection, and underlying data verification. TB: data interpretation and writing. HM: study design, data collection, data analysis, data interpretation, and writing.

## Conflict of Interest

TB is chairman of INDO Lighting Limited who manufacture a certified development version of the prototype respirator presented in this paper. The remaining authors declare that the research was conducted in the absence of any commercial or financial relationships that could be construed as a potential conflict of interest.

## Appendix

### Usability Questions

1. How comfortable was the PeRSo to wear?

2. How easy was it to perform basic tasks in the PeRSo?

3. How easy was it to don the PeRSo?

4. How easy was it to doff the PeRSo?

5. How was the patient's response to the PeRSo?

6. What did you not like about the PeRSo?

7. How would you rate the overall experience of wearing the PeRSo?

8. Any additional comments?
